# Perception of facing life's challenges in patients with spinal cord injury in Iran: a qualitative study

**DOI:** 10.1186/s40359-022-00909-2

**Published:** 2022-08-15

**Authors:** Fateme Mohammadi, Khodayar Oshvandi, Mostafa Bijani, Seyed Reza Borzou, Masoud khodaveisi, Seyedeh Zahra Masoumi

**Affiliations:** 1 Chronic Diseases (Home Care) Research Center and Autism Spectrum Disorders Research Center, Department of Nursing, Hamadan University of Medical Sciences, Hamadan, Iran; 2 Mother and Child Care Research Center, Hamadan University of Medical Sciences, Hamadan, Iran; 3 Department of Medical Surgical Nursing, Fasa University of Medical Sciences, Fasa, Iran; 4 Department of Medical-Surgical Nursing, School of Nursing and Midwifery, Chronic Diseases (Home Care) Research Center, Hamadan University of Medical Sciences, Hamadan, Iran; 5 Department of Community Health Nursing, School of Nursing and Midwifery, Chronic Diseases (Home Care) Research Center, Hamadan University of Medical Sciences, Hamadan, Iran; 6 Department of Midwifery, School of Nursing and Midwifery, Mother and Child Care Research Center, Hamadan University of Medical Sciences, Hamadan, Iran

**Keywords:** Patient, Spinal cord injuries, Life's challenges, Qualitative study

## Abstract

**Background:**

Spinal cord injury (SCI) is one of the most serious types of physical trauma and has become a major life-threatening condition in the recent decade. It is essential that the life perception and experiences of patients with SCI be studied and evaluated in different cultural contexts so that their needs and the challenges they face can be properly determined. The present study aims to explore the how patients with SCI in the south of Iran perceive facing life's challenges.

**Methods:**

The present study is a qualitative research with a descriptive phenomenological design. Participants were identified through purposive sampling of patients with spinal cord injury admitted to two state hospitals affiliated with a university of medical sciences in western Iran. The researchers collected data using semi-structured, in-depth interviews with 25 SCI patients conducted between August and October 2021. Data was analyzed according to Colaizzi's method using MAXQDA v. 2007.

**Results:**

Analysis of the data led to the emergence of three themes and nine sub-themes. The three main themes were emotional shock (crisis making and mental rumination, persistent depressive disorder, pitying behaviors, fear of the future), loss of dignity (poor self- care, sexual dysfunction, loss of job and educational status), and lack of effective support (lack of financial institutions and sponsors, lack of social support).

**Conclusion:**

Patients with spinal cord injuries face various issues in their care and social lives. Attention to their psycho-emotional needs along with comprehensive health support play key roles in generating a sense of self efficacy and promoting the mental well-being and dignity of patients with spinal cord injuries. Accordingly, healthcare administrators and caregivers are recommended to provide more comprehensive health support to SCI patients to meet their needs more effectively.

## Introduction

Spinal cord injury (SCI) is one of the most serious types of physical injury and is among the major life-threatening conditions of the recent decade [1, 2]. The World Health Organization (WHO) reports that 40 million people worldwide suffer from SCIs and nearly three million new cases emerge every year [3]. Spinal cord injuries often occur in young individuals, over 23% of SCIs occur in individuals aged between 19 and 30 years, and young active men are at higher risk [4]. Because men are very active at a young age and have more work and sports conflicts, they also tend to drive in a high-risk manner and at high speeds, which in turn can result in traumatic SCIs [4, 5]. In the U.S., there are currently over 300,000 individuals with SCIs, and 8,000 to 10,000 new cases are added to that number every year [5]. Because of the high rate of car accidents in and between cities and the war which lasted eight years, Iran also has many SCI cases; there are currently 100,000 known cases, and 3,000 new cases are recorded every year [6].

### Previous research on spinal cord injury patients’ needs

Over recent years, researchers have used qualitative approaches to identify the challenges faced by SCI patients in life, especially psycho-emotional issues, from their caregivers’ perspective [7, 8]. Zanini et al. [7] reported that depression and loneliness in SCI patients are outweighed by their feelings of inefficiency and poor self-confidence following the loss of their physical autonomy and financial independence and disorders in their emotional interactions and sexual relationships. Many SCI patients were active young adults with numerous social interactions whose lives changed dramatically with one incident in which they lost their ability to move their body parts and control their bodily functions voluntarily.

They have also seen their occupations, emotional interactions, and marital relationships suffer as a result, and they hold no hope of change [4, 6, 7, 9]. Some qualitative studies have examined the life challenges of SCI patients from the perspective of the patients themselves [8, 10–12]. Hall et al. reported that SCI patients experience many psychological, financial, environmental, and interactive challenges and require comprehensive physical and social support [8]. Duggan et al. also stated that the duration of spinal cord injury, financial status, and the level of support strongly affect the patient’s life experiences and quality of life [12].

The experiences of patients with spinal cord injuries are completely dependent on the socio-cultural context and environmental support (family, community, etc.). It is, therefore, essential that the life perception and experiences of patients with spinal cord injuries be studied in different cultural contexts so that their needs and problems are accurately identified and their living conditions improved. This study aimed to explore the way patients with spinal cord injuries in Iran perceive life.

## Methods

The present qualitative research with a descriptive phenomenological design [13] was conducted from August to October 2021. The reporting of the results was based on the consolidated criteria for reporting qualitative research (COREQ) checklist [14].

### Data collection

Patients with spinal cord injuries formed the sample of this study. The inclusion criteria comprised being willing to participate, having at least 12 months’ history of a spinal cord injury, being Iranian, having a good command of Farsi, and being able to provide adequate and appropriate information. The corresponding author acquired the phone numbers of 60 SCI patients from the Iranian SCI association and two state-owned hospitals affiliated with a university of medical sciences in western Iran. Of the forty-five patients who met the inclusion criteria, twenty-five patients agreed to participate in the study, while twenty patients expressed their refusal to participate in the first phone call made by the corresponding author, because of depression, fatigue, or time limitations. Participants, who were selected through purposeful sampling, were interviewed until data saturation was reached and no new categories emerged. In this study, data saturation was reached after 23 interviews, and two further interviews were done to ensure no new data was available.

Data was collected through 25 individual semi-structured interviews. The participants were interviewed through video calls on WhatsApp at times which were convenient for them. The corresponding author conducted and analyzed all the interviews inductively. Each interview began with a few general questions. Based on the respondents’ initial answers, follow-up questions were subsequently asked to increase the clarity of the information (Table 1). Each interview lasted between 45 and 75 min and was recorded with the verbal and written consent of the participant. Immediately after each interview, the corresponding author reviewed the recording several times to develop a general understanding and deeper insight. Then the recording was transcribed verbatim. Field notes were recorded as well. The main questions and subsequent probing questions are shown in Table 1.

**Table 1:** The main questions and their subsequent probing questions

General questions	Follow-up questions
Can you describe a typical day in your life?	Can you explain further?
Please describe the development of your illness?	Can you explain further? What do you mean by that?
What feelings did you experience during this period?	Can you give an example?
How has this incident affected your life?	Can you explain further?
What does living with a spinal cord injury mean to you?	What do you mean by that?

### Data analysis

Data was analyzed according to Colaizzi's method, which consists of seven steps: (1) reading and re-reading each transcribed interview, (2) extracting important terms and phrases from the transcripts, (3) assigning meaning to the extracted units, (4) organizing and categorizing similar units, (5) presenting comprehensive descriptions of the extracted categories, (6) creating a basic paradigm of the subject under study according to the extracted categories, and (7) confirming the basic paradigm by having the participants verify the themes and categories [15, 16]. MAXQDA v. 2007 was used for data analysis.

### Rigor

To increase the credibility and accuracy of the data, the researchers used a combination of methods: semi-structured interviews, prolonged engagement with the data, member checking, and peer checking. The extracted concepts and themes were presented to two of the participants and three peers who stated that the findings were consistent with their understanding and interpretations. Furthermore, the researchers bracketed all their prior information and personal beliefs about the care of these patients to avoid the influence and interference of personal beliefs. Finally, confirmability was achieved through accurate recording of the participants’ narratives and detailed reporting of the study results to provide the possibility of follow-up for other researchers.

## Results

**Fig. 1:**
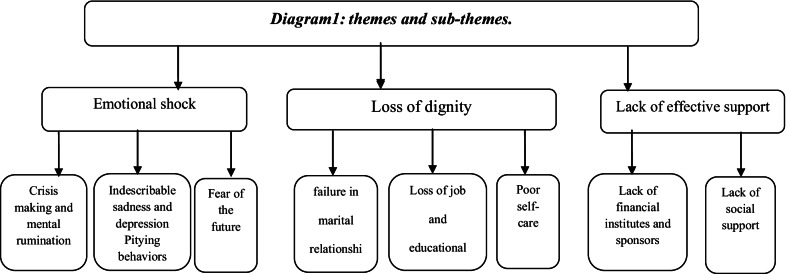
.

Out of the 25 patients, 15 were male and 10 were female. The mean of the patients’ ages was 39.45 ± 2.69. The majority of participants were single, and spinal cord injuries were caused by car accidents. Analysis of the qualitative data yielded three themes and nine sub-themes. Table 2 shows the demographic characteristics of the participants. The three main themes were emotional shock, loss of dignity, and lack of support. Figure 1 shows the themes and sub-themes.

**Table 2:** Individual characteristics of the participants

Participants	Gender	Age	Cause of injury	Marital status
P1	Male	29	Car Accident	Single
P2	Male	32	Car Accident	Single
P3	Male	44	Falling Down	Married
P4	Female	38	Car Accident	Single
P5	Male	26	Beat	Single
P6	Male	30	Car Accident	Single
P7	Female	48	Car Accident	Married
P8	Female	30	burst	Single
P9	Male	37	Car Accident	Single
P10	Female	41	Car Accident	Divorced
P11	Female	27	Falling Down	Single
P12	Male	41	Car Accident	Married
P13	Female	39	Car Accident	Single
P14	Male	51	Car Accident	Married
P15	Female	40	Car Accident	Single
P16	Male	42	Falling Down	Married
P17	Female	39	Car Accident	Divorced
P18	Male	37	Beat	Single
P19	Male	47	Falling Down	Married
P20	Male	46	Falling Down	Married
P21	Female	57	Car Accident	Married
P22	Male	39	burst	Single
P23	Male	33	Car Accident	Single
P24	Female	36	Falling Down	Married
P25	Male	31	Car Accident	Married

### Theme 1: emotional shock

One main theme extracted from the participants’ experiences was emotional shock, which consists of the following sub-themes: crisis making and mental rumination, indescribable sadness and depression, pitying behaviors, and fear of the future.

#### Crisis making and mental rumination

SCI patients described their condition as a horrible, painful, and unbearable crisis. Their perception of their condition as a crisis led to their mental rumination about the consequences of their injuries. Crisis making and the resulting mental rumination caused patients to lose all hope for the future, lose the ability to adapt effectively to their situation, and experience severe psychological issues including anxiety and chronic depression. According to one of the patients:

*“*My whole life has been ruined by this disaster. What can I do now in this situation? My life is filled with pain and suffering. All my hopes and dreams are gone. What use does my living have now? I wish for death every day. I can’t put up with this situation anymore” (Participant 5).

#### Indescribable sadness and depression

The experiences of SCI patients showed that spinal cord injuries aggravate the symptoms of depression. These patients stated that they were suffering from post-traumatic stress, insomnia, lethargy, despair, and guilt and were taking antidepressants and anti-anxiety drugs. One participant stated:

“What sin did I commit to deserve this catastrophe? I’m tired of this life. I’ve wanted to kill myself several times and put myself and my family out of this misery” (Participant 6).

Another patient stated:

“I have dreadful nightmares. I do not have any peace at all. I feel that my life is not worth living. I am of no use to anyone, and I’m just a burden on my family and society” (Participant 3).

#### Pitying behaviors

Pitying behaviors and undue expressions of sympathy on the part of their families, society, and caregivers cause patients with SCIs to feel inefficient and unvalued, which in turn makes them feel upset and angry. From the patients’ point of view, pitying looks and offers of help undermine their self-esteem and are viewed as an insult to their human dignity. According to one SCI patients:

“The pity my family and relatives show me really bothers me. I don’t want them to help me when they remind me of what they are doing for me. This kind of sympathy is such a pain. Of course, sometimes they really want to help, but they don’t know how to do it right, and that makes me upset. The way they help me is like the friendship of the bear and the gardener” (Participant 13).

#### Fear of the future

“Every day, I think about what the future holds for me. Is there any chance of my recovery so I can have a useful life and keep my family together? My life is in chaos, and I don’t know what will happen next” (Participant 19).

### Theme 2: loss of dignity

The theme of loss of dignity consists of the following sub-themes: poor self-care, sexual dysfunction, and loss of job and/or educational status.

#### Poor self-care

A major factor adversely affecting the quality of life and self-efficiency of SCI patients is poor self-care. The patients in this study stated that they sometimes even needed assistance with their basic needs, such as eating, getting dressed, and using the bathroom, their dependence on others and lack of autonomy leads to poor self-care and health status.

According to one SCI patient:

“Life in a wheelchair is an ordeal. It is really sad when you can’t take care of yourself and have to rely on other people for your smallest and simplest needs, like a puppet whose movements are controlled by others and can’t do anything on its own” (Participant 23).

#### Failure in marital relationships

All SCI patients, both male and female, expressed that in their experience, SCIs can result in failed marital relations, which adversely affects the patients’ marital relationships. These patients were not satisfied with their marital relationships and were not willing to engage in sexual acts with their spouses. In some cases, their failure in marital relations had led to divorce.

“I feel I’ve lost my masculinity. I have no desire for or interest in having sexual relations. I can’t satisfy my wife’s sexual needs. I suffer from premature ejaculation and can’t enjoy sex with my wife, which embarrasses me” (Participant 25).

Another SCI patient stated:

“I’m really worried about my life. My wife keeps saying that she wants to have a baby, but I hate sexual relations and have no desire for sex. I can’t help it. Before my spinal cord was injured, I had very intimate relationships with my wife and really enjoyed my sexual relationship with her, but now, I just don’t have any interest in having sex. Maybe it’s because of my injury. The day is approaching when my wife will divorce me because of our inability to have marital interactions” (Participant 7).

#### Loss of job and/or educational status

Another sub-theme of loss of dignity was loss of job and/or educational status. Spinal cord injuries can lead to many issues in the patients’ occupational, educational, and other social activities. One participant stated:

“Who would give a person with a spinal cord injury a job? In the eyes of society, we are useless and incapable people who can’t do anything. It’s not our fault. It’s not like we wanted this to happen to us. No one understands us” (Participant 14).

### Theme 3: lack of effective support

The final extracted theme was lack of effective support with the sub-themes of lack of financial institutions and sponsors and lack of social support. Based on the patients’ experiences, healthcare policy makers and administrators have not taken any effective measures to support patients with SCIs, and their needs have been mostly neglected. The patients mentioned that even such simple tasks as crossing the street or using public transportation was difficult for them and that no attempts had been made to satisfy their needs.

#### Lack of financial institutes and sponsors

Many of the patients in this study mentioned that there were only a few institutes or charities which helped them.

“In these difficult times with this high cost of living, how are we supposed to meet our needs? No one gives us a job, no one employs us, and we don’t get any financial support. What should we do then?” (Participant 15).

Another patient with SCI stated:

*“*The costs of treatment are exorbitant. Nobody feels responsible for us. It’s as if we didn’t belong to society. Why don’t the authorities do something for us? Where are these charities that they say will help us?” (Participant 11).

#### Lack of social support

The patients’ experiences showed that the authorities in healthcare and other departments, including city planning and transport, pay no attention to the rights of citizens with SCIs and have not taken any measures to create the necessary infrastructure for them.

“They just don’t care that people with spinal cord injuries live in the same society as others and have rights as citizens. They have the right to live too, and their human dignity should be respected” (Participant 9).

According to another patient:

“When we go to government departments to apply for social services, hasn’t anyone thought about how a person with a spinal cord injury should climb all those stairs to get from one level to another? How can we use elevators? Even crossing the street is an ordeal. There is no specific place for people like us to board public transportation. We can’t use social and municipal services such as parks and movie theaters like other people, because the facilities for us are lacking, and it’s hard to get around” (Participant 17).

## Discussion

The present study was conducted to investigate patients’ perception of living with spinal cord injuries in the south of Iran. Analysis of the lived experiences of SCI patients identified three themes: emotional shock, loss of dignity, and lack of effective support. One of the most significant experiences of the patients in the present study was emotional shock, which was comprised of crisis making and mental rumination, indescribable sadness and depression, pitying behaviors, and fear of the future. The emotional shock following the injury caused the patients to engage in crisis making and mental rumination. Each patient regarded their injury as a horrific disaster which had affected all aspects of their personal and social lives. They also mentioned that their spinal cord injuries had ruined their lives forever and that they had no hope of recovery. This crisis making led to their mental rumination. Nolen-Hoeksema [17] posits that rumination, as a perseverative, past-oriented, and passive way of negative thinking, puts individuals at risk of experiencing intensified and prolonged symptoms of emotional distress.

In the present study, mental rumination caused patients to consider all their dreams lost and to constantly think about the disaster that had befallen them. Their belief that all was lost for them prevented them from effectively adapting to their condition. Many studies have reported that spinal cord injuries can lead to severe psycho-emotional issues [18, 19], yet crisis making and mental rumination, which can contribute to psychological crises and emotional shock in patients with SCIs, have not been adequately discussed. It is, therefore, recommended that caregivers, especially psychologists and psychiatric nurses, identify their patients’ negative thoughts and help them avoid crisis making and mental rumination and the resulting psychological crises and emotional shock that will threaten their mental health.

Another sub-theme of emotional shock was indescribable sadness and depression, which was found to have caused the patients to experience feelings of despair, frustration, guilt, worthlessness, shame, embarrassment, and isolation. The intensity of depression and other psycho-emotional issues was so great in some patients that they had considered suicide.

In line with the present study, Tchajkova et al. [20] stated that SCI patients reported having suicidal thoughts within the first two years of experiencing their injury. However, no participants thought that they would have been able to make an informed decision about medical assistance-in-dying during this time. Bombardier et al. [21] also stated that patients with spinal cord injuries experience more anxiety and psychological tension and subsequently suicidal thoughts.

McCullumsmith et al. [22] said that SCI patients have reported many psychological attacks that are influenced by the level of environmental reward and control, spiritual well-being, and severity of SCI. Based on these results, the mentioned studies suggest that nurses, healthcare providers, and authorities employ effective educational interventions to increase psychological empowerment in patients with SCIs and to address their psychological issues [20–22].

Another sub-theme of emotional shock in the present study was pitying behavior. Even though expressing sympathy and empathy with patients is among the most important duties of professional healthcare providers, they should avoid pitying looks and behaviors and excessive sympathy so as to maintain their patients’ dignity and mental security [23]. Undue expressions of sympathy and pity disturb patients with SCIs, who consider such expressions to be an insult to their dignity. Studies report that sympathy and empathy with patients cause patients to feel better and deal with the stressful conditions of their illness more easily [24, 25], yet pitying patients has an adverse effect on their mental health and, by extension, self-confidence and psychological security, which results in feelings of worthlessness, incompetency, and frustration [26]. Studies of patients with hard-to-treat diseases show that to preserve the health and psychological security of these patients and their families, caregivers and the whole of society should treat them with respect and avoid stigmatizing them or pitying them [25, 27].

Fear of the future was another sub-theme of emotional shock in the present study. Participants expressed their feelings of indescribable and strange fear of the future. Even thinking about what the future held for them was terrifying for them. They worried about solidarity in their families, job security, financial status, and social interactions. In line with the present study, other studies have reported that patients with spinal cord injuries mention financial and family problems which cause them to fear the future and life with their SCI [28, 29]. Therefore, comprehensive family, social, and medical support as well as extensive education about the condition can prove useful in alleviating these patients' fear of the future.

Another theme extracted from the participants’ experiences was loss of dignity, which consists of poor self-care, sexual dysfunction, and loss of job and/or educational status. In SCI patients, poor self-care, lack of autonomy in taking care of oneself, and dependence on family members for self-care led to poor self-efficacy and self-management. Patients with spinal cord injuries have limitations in performing their daily activities; empowering these patients in self-care, however, can facilitate their recovery. In line with the present study, Carpenter et al. stated that rediscovering oneself, and creating a new identity are among the most important challenges for SCI patients which play important roles in the mental status and quality of life of these patients [30]. Amann et al. [11] also reported that empowering patients with SCIs in self-care and multidisciplinary and comprehensive rehabilitation care can make a significant contribution to the patients’ self-efficacy, which will help them prevent further physical injuries and improve their psychological health and security. It can also create a sense of autonomy and self-sufficiency in the patients and encourage them to play a more active part in their personal and social activities. Another sub-theme of loss of dignity was sexual dysfunction. The present study found that the participants suffered from issues in their marital sexual relations, which is consistent with the findings of Moghimian et al. [31]. It appears that the care plans of SCI patients should include more attention on sexual hygiene and health. On a similar note, the results of the study of Taylan et al. [32] in Turkey showed that spinal cord injuries adversely affect the sexual life of SCI patients, leaving them feeling hopeless in dealing with their sexual issues. Loss of libido, premature ejaculation, and fear of sexual relationships are among the issues reported by Taylan et al., which is consistent with the findings of the present study.

Occupational or educational incompetency was another sub-theme of loss of dignity. Spinal cord injuries can threaten patients’ job prospects, education, and other social activities. Other studies have referred to the occupational and educational issues of patients with SCIs [33], but the lack of facilities and inadequate social support for this population in Iran made the patients in the present study experience more difficulties in their jobs or education, which is consistent with the findings of Babamohamadi et al. [34].

The last theme extracted from the participants’ experiences was lack of effective support, which is comprised of lack of financial institutions and sponsors and lack of social support. Healthcare policymakers and administrators must develop effective practical plans to support individuals with spinal cord injuries and to address their healthcare needs.

Inadequate support from charities, government departments, and healthcare organizations, the high costs of treatment, disregard for their rights as citizens, and the lack of special facilities in city and office buildings are the most important issues extracted from the participants’ experiences with regard to effective support. The participants mentioned that they faced problems in receiving even the most basic services, including transportation, public amenities (e.g. parks and movie theaters), and education. Similarly, the study of Atobatele [35] found that patients with SCIs find it difficult to use public amenities such as transportation and other social services, e.g. those provided by public offices. In explanation of this issue, it can be said that in the less-or moderately-developed countries, the lack of facilities, economic problems, and a lack of infrastructure exacerbate these problems more than in developed countries.

According to the study of Islam et al. [36] in Bangladesh, the costs of treatment and rehabilitation programs are too high for patients with SCIs, many of whom face poverty as a result of their injury, and the support given by authorities and charities is not adequate. In the present study, the participants believed that effective social and financial support and easy access to facilities provided by social organizations affiliated with the government and private foundations can reduce their anxiety and, consequently, improve their psychological security. Patients with SCIs can have psychological security only when they are given comprehensive support combined with respect for their human dignity. Similarly, other studies have reported that social organizations and foundations which support hospitalized patients and other vulnerable groups in the society play a major role in increasing the psychological security of these populations [37].

### Limitations

No external people transcribed the interviews in the present study. Therefore, it is suggested that in future studies, the data be transcribed by an external individual. Moreover, SCI patients preferred online interviews because of physical and motor problems and the prevalence of COVID-19. Therefore, face-to-face interviews should be conducted in future studies to collect more accurate information.

### Strengths

The present study is the first qualitative attempt at investigating the lived experiences of patients with SCIs in Iran and is an innovation in this regard. As the cultural, economic, and social conditions in less-or moderately-developed countries, including Iran, are different from developed countries, it is suggested that researchers conduct similar studies in other countries.

## Conclusion

The life experiences of patients with SCIs are complex and completely dependent on culture. SCI patients in Iranian society experience many challenges and emotional problems following the loss of their job and/or educational position in the family. In this regard, extensive support for these patients as well as empowering them in efficient self-management can affect their experiences and their view of life. Accordingly, it is necessary to create a cultural, professional, and organizational context in which this population can receive proper care and their psychological security is maintained. Healthcare policy makers and administrators are recommended to use the findings of the present study to provide more comprehensive healthcare services and support to this group of patients.

## Data Availability

The datasets generated and/or analysed during the current study are not publicly available due to the necessity to ensure participant confidentiality policies and laws of the country but are available from the corresponding author on reasonable request.

## Abbreviation

WHO: World Health Organization
